# Green Tea Catechins Attenuate Neurodegenerative Diseases and Cognitive Deficits

**DOI:** 10.3390/molecules27217604

**Published:** 2022-11-06

**Authors:** Obaid Afzal, Mahmood Hassan Dalhat, Abdulmalik S. A. Altamimi, Rabia Rasool, Sami I. Alzarea, Waleed Hassan Almalki, Bibi Nazia Murtaza, Saima Iftikhar, Shamaila Nadeem, Muhammad Shahid Nadeem, Imran Kazmi

**Affiliations:** 1 Department of Pharmaceutical Chemistry, College of Pharmacy, Prince Sattam Bin Abdulaziz University, Al-Kharj 11942, Saudi Arabia; 2 Department of Biochemistry, Faculty of Science, King Abdulaziz University, Jeddah 21589, Saudi Arabia; 3 Institute of Molecular Biology and Biotechnology, The University of Lahore, Lahore 54000, Pakistan; 4 Department of Pharmacology, College of Pharmacy, Jouf University, Aljouf, Sakaka 72341, Saudi Arabia; 5 Department of Pharmacology, College of Pharmacy, Umm Al-Qura University, Makkah 21955, Saudi Arabia; 6 Department of Zoology, Abbottabad University of Science and Technology (AUST), Abbottabad 22310, Pakistan; 7 School of Biological Sciences, University of the Punjab, Lahore 54000, Pakistan; 8 Department of Zoology, Kinnaird College for Women, 93-Jail Road Lahore, Lahore 54000, Pakistan

**Keywords:** catechins, neuroprotective, neurodegenerative diseases, epigallocatechin gallate, cognitive defect

## Abstract

Neurodegenerative diseases exert an overwhelming socioeconomic burden all around the globe. They are mainly characterized by modified protein accumulation that might trigger various biological responses, including oxidative stress, inflammation, regulation of signaling pathways, and excitotoxicity. These disorders have been widely studied during the last decade in the hopes of developing symptom-oriented therapeutics. However, no definitive cure has yet been discovered. Tea is one of the world’s most popular beverages. The same plant, *Camellia Sinensis* (L.).O. Kuntze, is used to make green, black, and oolong teas. Green tea has been most thoroughly studied because of its anti-cancer, anti-obesity, antidiabetic, anti-inflammatory, and neuroprotective properties. The beneficial effect of consumption of tea on neurodegenerative disorders has been reported in several human interventional and observational studies. The polyphenolic compounds found in green tea, known as catechins, have been demonstrated to have many therapeutic effects. They can help in preventing and, somehow, treating neurodegenerative diseases. Catechins show anti-inflammatory as well as antioxidant effects via blocking cytokines’ excessive production and inflammatory pathways, as well as chelating metal ions and free radical scavenging. They may inhibit tau protein phosphorylation, amyloid beta aggregation, and release of apoptotic proteins. They can also lower alpha-synuclein levels and boost dopamine levels. All these factors have the potential to affect neurodegenerative disorders. This review will examine catechins’ neuroprotective effects by highlighting their biological, pharmacological, antioxidant, and metal chelation abilities, with a focus on their ability to activate diverse cellular pathways in the brain. This review also points out the mechanisms of catechins in various neurodegenerative and cognitive diseases, including Alzheimer’s, Parkinson’s, multiple sclerosis, and cognitive deficit.

## 1. Introduction

**Figure 1:**
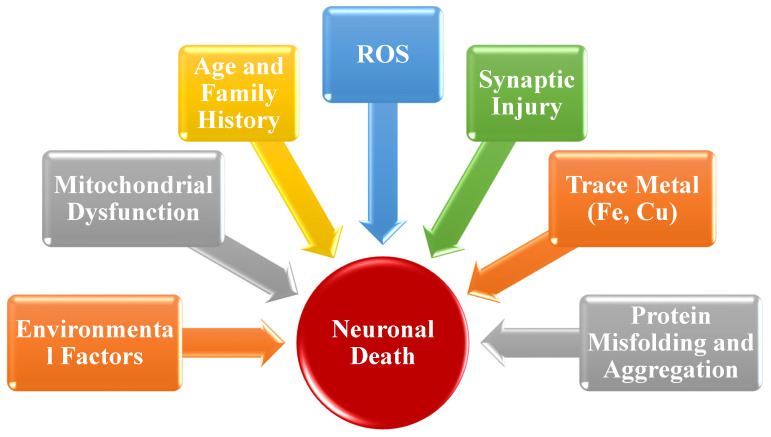
Pathophysiological aspects that can cause neurodegeneration.

Neurodegenerative diseases (NDs) are one of the most prevalent health problems, affecting millions of people all around the world [1]. Because of the aging population, longer life, and changing environments, the worldwide burden of NDs is increasing. According to the World Health Organization (WHO), NDs account for 12% of total world mortality, 16.8% of deaths in developing countries, and 13% in developed countries. It is estimated that, by 2030, neurological disorders, such as Alzheimer’s disease (AD), Parkinson’s disease (PD), and other dementias, will constitute 38% of worldwide disability as calculated by years of life lost owing to disability [2]. According to the WHO, the global cost of dementia was estimated to be USD 818 billion in 2015, which was approximately 1.1% of the global gross domestic product (GDP) [3]. The NDs, including AD, PD, multiple sclerosis (MS), Huntington’s disease (HD), and amyotrophic lateral sclerosis (ALS), are caused by diverse etiological causative factors, such as genetic, molecular, and environmental factors. Inflammation, excessive reactive oxygen species (ROS), epigenetic instability, and aging are the primary determinants of neurodegenerative diseases. The molecular factors involved in NDs are (1) particular protein dynamics coupled to degradation and aggregation of the impaired protein, (2) formation of free radicals and oxidative stress, (3) mitochondrial dysfunction and deficient bioenergetics, and (4) exposure to pesticides and metal toxicity [4], as shown in Figure 1.

Neuronal deterioration accelerates with age and is particularly severe in old age, as observed with the numerous neuropathologies. To date, most neurodegenerative diseases have remained incurable. As previously stated, the current ongoing treatment just relieves symptoms but does not hinder the disease progression, focusing the need for more effective treatment strategies [5]. Development of new drugs for NDs is hampered by the lack of understanding of the biological perspective of these multifactorial disorders, the blood–brain barrier that prevents drug flow to the brain, and the scarcity of clinically relevant animal models for new drug testing [6]. Although NDs are pathologically characterized by disease-specific misfolded protein aggregation and modified cellular stress response, researchers have focused exclusively on reduction in misfolded protein load; however, the outcomes were disappointing [7,8]. Due to the late onset and asymptomatic nature of most neurodegenerative diseases, treatment begins at later disease stages and has restricted benefits to the patients. If interventions are initiated early, they might be able to largely prevent or slow the disease progression. These therapies via lowering or even removing the primary stressor may be able to restore neuronal function [9].

Flavonoids are primary molecules for establishment of a new class of clinically effective therapeutic agents for neurodegenerative disorders. Flavonoids have been linked to a decreased risk of neurodegenerative disorders when consumed regularly [10]. Besides their antioxidant properties, these polyphenolic compounds have neuroprotective characteristics due to their ability to interact with cellular signaling pathways, followed by translation and transcription, which influence cellular function in both physiological and pathological conditions [8,9]. The most popular beverage used throughout the world, tea, can be divided into six categories based on the degree of fermentation, such as non-fermented form (green tea), white tea (micro-fermented), yellow tea (slightly fermented), oolong tea (semi-fermented), black tea (fully fermented), and dark tea (post-fermented) [11,12,13]. Tea includes a wide range of bioactive compounds, including flavonoids, caffeine, polyphenols, free amino acids, theanine, methylxanthine, several lipids, volatile molecules, as well as mineral substances [14,15,16,17]. Green tea usually contains a higher content of polyphenols than black tea due to polyphenol biodegradation during processing of dark tea, which has beneficial effects on human health [15].

Catechins, also known as flavan-3-ols, constitute about 70–80% of tea polyphenols and are in abundance in the young tea plant’s buds and leaves [18,19]. Meanwhile, green tea has stronger antioxidant activity in vitro as compared to dark tea. Dark tea, on the other hand, frequently exhibits greater in vivo antioxidant activity as compared to green tea due to the bioavailability of polyphenol biodegradation products [12,20]. Numerous studies have demonstrated that tea has a variety of health benefits, including memory and cognitive improvements, cardiovascular disease prevention, anti-cancerous, anti-obesity, and sedative [21,22,23,24,25]. The effect of tea on neuron function has generated great attention over the last few decades, and several studies have proved its neuroprotective properties. For instance, tea may help in reducing the morbidity and severity of Alzheimer’s and Parkinson’s disease, as well as the risk of developing depression symptoms via reducing inflammation and oxidative stress, regulating intracellular signaling pathways, metal chelation, and modulating the hypothalamus–pituitary–adrenal (HPA) axis and monoamine neurotransmitter levels [26,27,28]. Epigallocatechin gallate (EGCG) was able to attenuate any deficiencies in nest building and Barnes maze performance [29]. Moreover, EGCG has been shown to ameliorate central memory deficit in water mazes and novel object recognition tests [30]. EGCG also protects the majority of motor deficits induced by rotenone in grip strength measurement, Rota rod, beam-crossing task, and open-field test [31]. EGCG-loaded nanoparticles show promising application potential due to their degradability and biocompatibility [32]. It was evident that EGCG-loaded nanoparticles ameliorate neurobehavioral deficits in novel object recognition, open-field, and Morris water maze test [33].

## 2. Catechins: Biosynthesis and Mechanism of Action

### 2.1. Synthesis and Structure of Catechins

**Figure 2:**
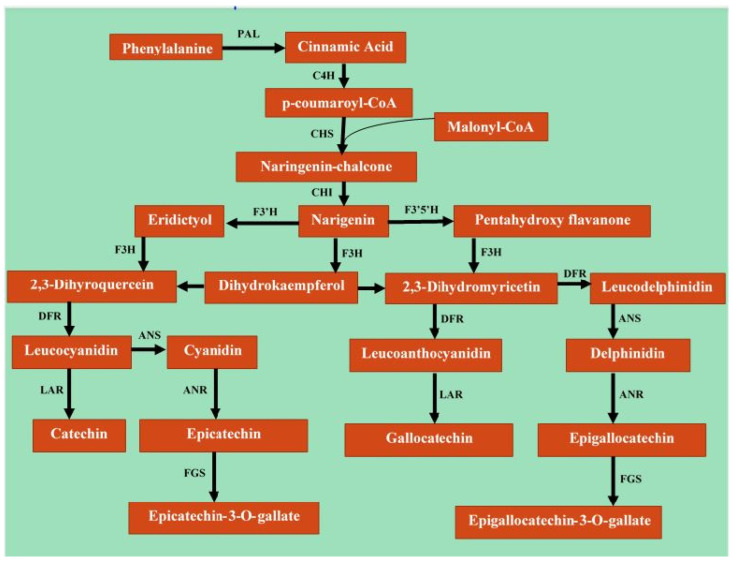
Biosynthesis of catechins in *Camellia sinensis* plant’s leaves via shikimic–cinnamic acid and acetic–malonic metabolic pathways. PAL, phenylalanine ammonia-lyase; C4H, cinnamate 4-hydroxylase; CHS, chalcone synthase; CHI, chalcone isomerase; F3H, flavanone 3-hydroxylase; F3′H, flavonoid 3′-hydroxylase; F3′5′H, flavonoid 3′,5′-hydroxylase; DFR, dihydroflavonol 4-Reductase; LAR, leucocyanidin reductase; ANR, anthocyanidin reductase; ANS, anthocyanidin synthase; FGS, flavan-3-ol gallate synthase.

Catechins are formed of a three-carbon core coupled to two phenolic nuclei (aromatic rings) with many hydroxyl groups [34,35]. The catechins in tea are categorized into two groups known as epistructured catechins and non-epistructured catechins [36]. Epistructured catechins are the major tea catechins. Epigallocatechin gallate (EGCG) is the most abundant catechin in green tea, followed by epigallocatechin (EGC), epicatechin gallate (ECG), and epicatechin (EC). Non-epistructured catechins include gallocatechin (GC), gallocatechin gallate (GCG), catechins (C), and catechins gallate (CG) and are, on the other hand, only found in trace amounts in tea [37]. Catechins are produced in *Camellia sinensis* plant’s leaves via shikimic–cinnamic acid and acetic–malonic metabolic pathways (Figure 2). The shikimic acid pathways form chalcone and gallic acid, which ultimately synthesize various catechins [38]. The content of particular catechins in a fresh leaf of tea varies depending on the region of cultivation, nutrition of the plant, variety, kind of leaves (young or coarse), and time of the year. Frequently, the catechins profile in the extract of green tea leaf is comprised of EGCG 10–15%, EGC 6–10%, ECG 2–3%, and epicatechins 2% [39].

### 2.2. Physical and Chemical Properties of Catechins

**Figure 3:**
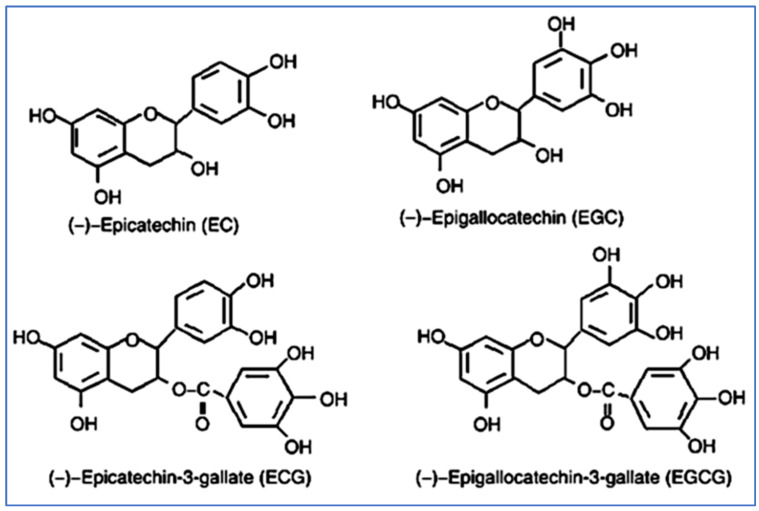
Different types of catechins.

Catechins are colorless, but they have bitter and astringent flavors [40]. A variety of catechins have distinct flavors, such as ECG and EGCG (Figure 3), which are bitter and astringent in taste, while EC and EGC have a bitter but sweet aftertaste [41,42]. Catechins can combine with proteins and caffeine to form precipitates, making the solution cloudy, known as cream formation [43]. Moreover, catechins precipitate when interacting with enzymes, including lipoxygenase, pepsin, amylase, lipase, and trypsin, thereby inhibiting their activity. Catechins with ester bonds, such as ECG and EGCG, have a higher ability to produce precipitates with enzyme interaction as compared to catechins containing non-ester bonds.

Isolation of catechins for their use in food can be achieved by precipitating catechins with protein or caffeine. Catechins have a strong iron-binging ability as the galloyl group in their structure binds to iron in food, preventing its absorption in the body [44]. Tea catechins are highly reactive and unstable in the presence of heat, oxidizing enzymes, and alkaline conditions. The oxidized catechins produce thearubegins and theaflavins in the presence of peroxidase (POD) and polyphenol oxidase (PPO) enzymes [45]. At 40 °C and a pH of 5.5, POD and PPO activity are optimal [46]. As a result, adjusting the temperature and pH can slow the activity of both these enzymes [47]. Temperature adjustment requires caution because epistructured catechins could epimerize to non-epistructured catechins at a high temperature above 95 °C. Furthermore, even though catechins are extremely stable in acidic solution (pH 4), their stability decreases with the rise in pH from 4 to 8, and catechins become highly unstable above pH 8 in alkaline solution. These qualities can be used to stabilize the catechins when added to foods. Due to their strong antioxidative properties because of hydroxyl groups in their structure, the catechins can scavenge reactive oxygen species (ROS), including superoxide radicals, nitric acid, hydroxyl radicals, peroxynitrite, singlet oxygen, and nitrogen dioxide, all contributing important roles in carcinogenesis [48]. The scavenging ability of hydroxyl radical (HO) declines in order of ECG, EC, EGCG, and EGC [49]. Furthermore, catechins have the ability to capture peroxyl radicals, hindering free radical chain reactions and halting lipid peroxidation [50]. The efficiency of four primary catechins in reducing lipid peroxidation decreases in the following order: EGCG, ECG, EGC, and EC [49]. This characteristic is crucial while adding catechins to foods, particularly those rich in oil or fat.

### 2.3. Bioavailability of Catechin

Depending on adsorption, distribution, metabolism, and excretion processes (ADME), the disposition of EGCG is at its peak at 90 min and is nearly undetectable after 24 h of its oral intake [51]. After oral administration, the EGCG reaches the stomach. The acidic environment of the stomach favors its structural stability [52]. Then, the portion of EGCG becomes absorbed in the small intestines. A minute EGCG concentration seems to be observed in peripheral blood because of the large fraction of EGCG in small intestines transmitted to large intestines, which causes the transformation of this molecule via enterocytes, forming approximately eleven catechin ring fission products. These ringed fission products are present conjugated as well as free form in plasma. The EGCG forms could cross the blood–brain barrier and reach brain parenchyma, promoting neuritogenesis and ultimately reducing neurodegeneration [53]. The remaining EGCG is metabolized via liver cells and converted into sulfated, glucuronide, and methylated intermediates, which are excreted in urine [54]. Several factors are involved in maintenance of polyphenols’ stability [51,55]. The alkaline environment and high temperature affect structural stability, enhancing chemical EGCG degradation [56]. Many studies hypothesized that, in humans, the oral bioavailability of EGCG is low and decreases along with food [57]. Some studies have reported selectivity of EGCG with different vitamins and minerals, suggesting that vitamins and fish oil reduce EGCG oxidation, while minerals, such as chrome and selenium, improve bioavailability and antioxidant activities [58,59,60]. Andreu-Fernandez et al. [61] suggested that the EGCG concentration in plasma is highest after Teavigo intake following overnight fasting, while the EGCG, when taken along with food supplements, improves the stability inside the body.

### 2.4. Catechins and Neurodegeneration

As low-molecular-weight (MW) secondary metabolites, flavonoids are present in vascular plants and are grouped into flavonols, flavones, flavan-3-ol, flavanones, anthocyanins, and isoflavones. The three-ring structure of all phenolic compounds with a 15-carbon skeleton includes two six-carbon benzene rings connected by a heterocyclic ring of pyran or pyrone. Further, 2-Phenyl-3,4-dihydro-2H-chromen-3-ol serves as the backbone of flavan-3-ols, the catechins. The catechins groups include epicatechins, catechins, epicatechins gallate (ECG), epigallocatechin gallate (EGCG), and gallocatechin (GC) [62]. About 60% of catechins in green tea are made up of EGCG, comprising a galloyl group, a tetrahydropyran moiety, a pyrogallol ring, and a benzenediol ring [63]. The chemical structure of the molecule will determine its biological function. The interface of the molecule in the biological matrix is influenced by the quantity of hydroxyl groups and their position [64]. It is demonstrated that EGCG has a greater antioxidative effect than EC or EGC due to its higher hydroxyl content [65]. Moreover, EGCG possesses two structures, 4-keto,3-hydroxyl or 4-keto, 5-hydroxyl moiety, and ortho-3′,4′-dihydroxy moiety, enabling chelation and neutralization of the metal ions [66]. Therefore, the use of catechins, especially EGCG, may be effective in treatment of certain neurodegenerative diseases.

#### 2.4.1. Anti-Inflammatory and Antioxidant Activity

Catechins’ most well-known biological actions are their free radical scavenging and antioxidant characteristics. The cellular components, such as protein, nucleic acids, DNA, and lipids, due to excessive ROS production and accumulation of defective mitochondria in brain cells, have been linked to neurodegenerative diseases, such as AD [67]. Catechins diminish lipid peroxidation [68]. As free scavengers, the phenolic hydroxyl groups have antioxidant characteristics, preventing new free radical production and regulation of protein synthesis associated with redox balance, such as maintenance of superoxide dismutase, catalase, and Nicotinamide dioxide phosphate (NADPH) [69]. By phosphorylating the p38 MAPK kinase and ERK 1/2 signaling pathways, the catechins increase the nuclear factor erythroid 2-related factor-2 (NRF2) activity [70]. Epigallocatechin gallate boosts the Nrf2 levels that are decreased in the hippocampus of patients suffering from Alzheimer’s disease and enhances expression of HO-1, which modulates cellular response towards oxidative stress, thereby protecting against cellular death [71,72]. Catechins modulate neuro-inflammation, which is characterized by microglial activation, through immunomodulatory characteristics. They also have a significant impact on neurodegenerative disease progression, including AD, HD, PD, and amyotrophic lateral sclerosis [73]. When compared with controls, co-cultures of the main catechins in green tea and lipopolysaccharide (LPS) significantly decreased the levels of interleukin-6 (IL-6) and tumor necrosis factor (TNF-α) in human neutrophils. Catechins increase levels of Nrf2 mRNA and anti-oxidative activities in activated B cells and decrease the expression of nuclear factor kappa light chain enhancer of activated B cell (NFB) protein and toll-like receptor 4 (TLR4) [74]. In in vitro LPS-induced neuro-inflammatory models, EGCG decreases nitric oxide (NO) and TNF-α production and reverses motor impairment [75]. Four months of EGCG consumption in APP/PS1 transgenic mice mitigated amyloid (A) plaques in the hippocampus and prevented activation of microglia. Along with higher levels of anti-inflammatory cytokines IL-10 and IL-13, lower levels of IL-1 were also observed [76]. These results suggested that catechins protect neurons via diminishing inflammatory mediators production through NFB, Nrf2, and TLR4/NFB pathways, as well as altering microglial activation. Interestingly, due to the iron-chelating characteristics of catechins, the iron deposition in neurons and microglia is inhibited, which might be due to ferroprotein 1 (FPN1) and divalent metal transporter 1 (DMT1) [77]. People who have neurodegenerative diseases have toxic aggregates of AB due to iron, which accumulate in their brains [78]. As a result, EGCG may affect AB levels by destabilising AB plaques, preventing aggregation of hyperphosphorylated tau or inhibiting translation of the amyloid precursor protein (APP) due to a decrease in labile fe2+ [79].

#### 2.4.2. Autophagic and Neuritogenic Activity

The neuritogenic activity of EGCG and its metabolites was observed in human neuroblastoma SH-SY5Y cells [80]. In PCl2 (TrkB) cells, a very minute concentration of EGCG (0.5M) and unfractionated green tea polyphenols enhance the neurotrophic potential of brain-derived neurotrophic factor (BDNF) [81,82], whereas the in vivo findings are yet to be confirmed. Autophagy is linked to aging, which protects neural cells by removing protein aggregates and degrading obsolete cell structures [83,84]. Protein aggregates play a significant role in neurodegenerative diseases. Catechin via different mechanisms, including transcription factor EB (TFEB), mTOR (molecular target of rapamycin), and 5′ AMP-activated protein kinase (AMPK), may affect autophagy [85,86]. By suppressing the expression of the DNA methyltransferase 2 (DNMT2) gene, Khali et al. [87] have reported upregulated expression of autophagal genes (Atg5 and LC3) in response to EGCG, both in in vivo and in vitro studies. Interestingly, continuous EGCG treatment reduces neuronal apoptosis in the hippocampal CA1 region, thereby reducing the learning and memory deficit in rats subjected to chronic unexpected mild stress. The decrease in apoptotic cells, soluble and insoluble A1, and restoration of autophagic flux in the hippocampus CA1 region of the stressed rats may be the cause of this improvement [88]. Catechins also increase beclin-1, which regulates autophagy and endocytosis. Catechins also have neuroprotective effects via protein kinase C signaling through reducing Bax, caspase-3, Bad, and poly(ADP-ribose) polymerase (PARP) [89,90,91].

#### 2.4.3. Dual-Specific Tyrosine Phosphorylation Regulated Kinase 1a Inhibition

EGCG has demonstrated an antagonist of a serine/threonine kinase known as dual-specificity tyrosine phosphorylation-regulated kinase (DYRK1A). DYRK1A is found on 21q22.2 loci of chromosome 21. DYRK1A is considered a substantial contributor to cognitive dysfunction because of its role in neuron differentiation, neurogenesis, synaptic plasticity, and cell death [92]. In mice overexpressing DYRK1A, EGCG-enriched supplements neutralize plasma and neuronal markers (NFkB and BDNF). Moreover, EGCG is safer for heart and liver function and also crosses the blood–brain barrier (BBB) [93].

### 2.5. The Role of Catechins in Various Neurodegenerative Disorders

Over the years, research on the molecular mechanism of catechins indicates their noteworthy potential in prevention and management of neurodegenerative diseases. The neuroprotective effect of catechins in various neurodegenerative diseases has been listed in Table 1.

#### 2.5.1. Alzheimer’s Disease (AD)

**Figure 4:**
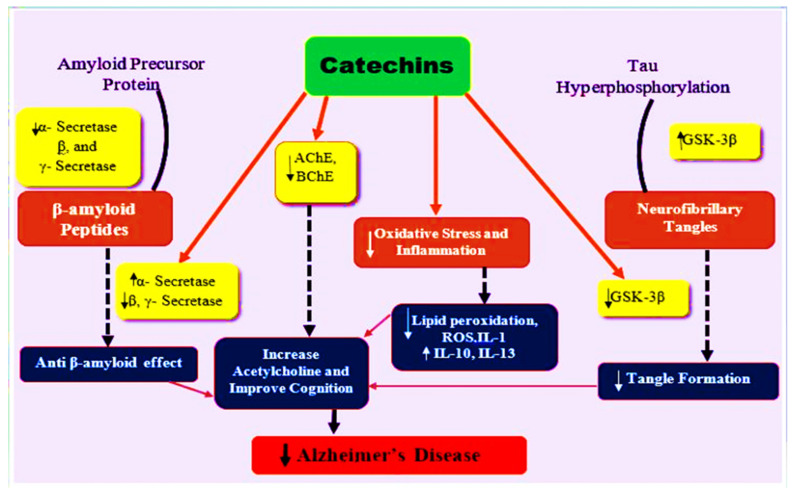
The neuroprotective mechanism of catechins in Alzheimer’s disease. A, β, and γ-secretase produce β-amyloid from amyloid precursor proteins, while GSK-3β catalyzes tau hyperphosphorylation that leads to neurofibrillary tangles, which ultimately results in oxidative stress and inflammation. The catechins inhibit β, γ-secretase, and GSK-3β activity, thereby preventing amyloid plaques and neurofibrillary tangles formation, as well as possessing anti-inflammatory and anti-oxidative properties and increasing acetylcholine levels in the synaptic cleft, thereby improving the decline in cognitive function in AD.

Numerous in vivo, in vitro, and in silico studies have been conducted on the molecular mechanism of catechins [91,107,108,109]. Antioxidant properties of catechins may help protect against neurodegeneration. Increased oxidative stress is linked to late-onset neurodegenerative diseases, as previously stated [110,111]. Peroxidized lipids, proteins, and oxidized DNA have all been found to be higher in Alzheimer’s disease patients [112]. Green tea catechins (0.5 percent green tea catechins in water) were given to rats for 26 weeks and were found to prevent amyloid-induced cognitive impairment. Both hippocampal and plasma levels of lipid peroxide and ROS were decreased by 20% as compared to controls [113]. EGCG was also shown to have a similar effect by Biasibetti et al. [114]. The researchers evaluated the effect of EGCG in a streptozotocin-induced dementia rat model. Cognitive deficits determined via the Morris water maze were reversed after one-month oral administration of EGCG (10 mg/kg/day). Moreover, the ROS and NO levels were dramatically reduced [114]. These antioxidative boosts may be due to the radical scavenging activity and iron-chelating abilities of catechins [115,116,117]. Catechins can chelate metal ions, such as copper and iron, by blocking the Fenton reaction [118] as these metal ions have been found to accumulate in people suffering from Alzheimer’s’ disease [119]. These outcomes demonstrate that catechins can attenuate the oxidative stress in the brain and peripheral tissues as well as prevent cognitive-deficit-related behavioral changes. Pro-inflammatory components, such as cytokines and cytotoxic compounds, are secreted from injured and damaged neurons, which could lead to cell death [120]. Lee et al. [121] demonstrated that EGCG pretreatment (1.5/3 mg/kg for 3 weeks) inhibits LPS-induced cognitive deficit and quashed cytokines and inflammatory proteins. Another in vivo study reported that EGCG reduced responses associated with LPS-induced inflammation in BV-2 microglia [122]. In Alzheimer’s’ disease, catechins affect the PKC-related pathways that are involved in cell survival and production of soluble non-toxic amyloid β [123,124]. Levtes et al. [125] found that low EGCG concentration (1–5 µM) induces production of sAPP from PC12 cells and human neuroblastoma, and 2 weeks of oral EGCG administration (2 mg/kg/day) enhances PKC α and ε in the hippocampus of mice as compared to controlled mice. Kaur et al. [126] and Kim et al. [127] investigated AchE inhibition with tea polyphenols. Kaur et al. [126] conducted research on aged Wistar rats fed green tea extract (0.5%) for 8 weeks and have shown significantly better learning and memory performance (measured through a passive avoidance test). Cerebrum’s AchE activity seemed reduced in older rats as compared to young rats. Kim et al. [127] reported the reversal of scopolamine-induced amnesia by (0.2% tea polyphenol) diet-based treatment. Tea polyphenols decrease AchE activity in addition to behavioral changes. Besides in vitro and in vivo studies, many in silico studies have also been carried out on tea polyphenol and acetylcholine esterase enzymes [109,128]. Hyperphosphorylation of tau protein and excessive neuronal β-amyloid deposition, inflammation, and oxidative stress are considered to be key contributors to development of AD. Green tea catechins possess a neuroprotective effect against Alzheimer’s disease by increasing anti-oxidant activity and inhibiting inflammation, oxidative stress, and hampering the action of beta-amyloid aggregation and anticholinesterase and other mechanisms, as shown in Figure 4.

#### 2.5.2. Parkinson’s Disease

Parkinson’s disease is a slowly progressive neurological condition of the motor system that typically affects people above the age of 50. However, people under 50 can also be affected. A-synuclein is a protein containing 140 amino acids, discovered in the brain, mostly expressed in the pre-synaptic cleft of nerve cells, involved in neuronal differentiation, regulation of dopamine synthesis, and suppression of neuronal apoptosis. In normal physiological conditions, α-synuclein cannot form a fibrillary structure due to the balance between its monomeric and oligomeric form. Furthermore, ubiquitin-proteasome machinery and lysosomal autophagic pathways remove the excess α-synuclein formed [129]. However, if α-synuclein levels are increased, there occurs impaired mitochondrial function, and its disruption with the membrane increases the predisposition of this non-toxic structure to aggregate and cause disruption in the normal neuronal mechanism, leading to neuron death [130,131,132]. Preventing aggregation formation is thus a vital step in preventing PD pathogenesis. The green tea polyphenol epigallocatechin-3-gallate (EGCG) aids in decreasing α-synuclein aggregation and toxicity in in vitro PCl2 cells. It also becomes attached to natively unfolded synuclein polypeptide chains to prevent formation of synuclein by forming an unstructured oligomer. It also impedes monomeric synuclein in addition to fibrillary intermediates [133].

**Figure 5:**
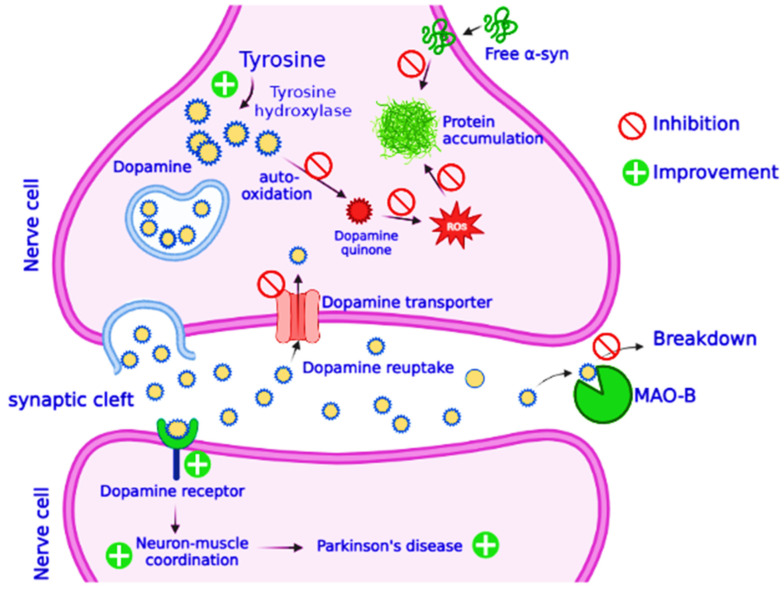
Role of green tea catechins in prevention of Parkinson’s disease. The protective mechanism of catechins has been elaborated. Parkinson’s disease is characterized by dopamine deficiency, and all treatment measures are aimed at introduction of dopamine as a medicine and its preservation. The figure illustrates conversion of tyrosine to dopamine by the action of an enzyme tyrosine hydroxylase; newly synthesized dopamine is packed into vesicles and released into the synaptic cleft, where it binds with the dopamine-specific receptors and contributes its role in signaling between two nerve cells. Normally, dopamine is reabsorbed to the nerve cells and reused, or it is broken down by the action of an enzyme monoamine oxidase B (MAO-B). Presence of green tea catechins inhibits MAO-B activity and promotes dopamine reabsorption. Catechins also inhibit plaque formation by the proteins and autoxidation of dopamine.

PD affects dopaminergic neurons in the Substantia Nigra pars compacta (SNpc) region of the brain [134]. According to an estimate, 80% of dopaminergic neurons die during PD, reducing the levels of dopamine in the brain [135]. Dopamine deficiency produces aberrant nerve firing and motor control loss [136]. Neurotoxin 1-methyl-4-phenyl-1,2,3,6-tetrahydopyridine (MPTP) causes dopaminergic neuronal death in substantia nigra and is widely used in discovering the molecular mechanism involved in pathogenesis of PD [137]. In cynomolgus monkeys given MPTP, catechins-rich polyphenol extract alleviated motor deficit, recovered tyrosine hydroxylase and dopamine levels, and reduced α-synuclein oligomers and their aggregation [138]. Pre-treatments with green tea extracts and EGCG reduced the loss of dopamine in male C57/BL mice by significantly altering striatal antioxidants, such as superoxide dismutase (SOD) and catalase. They also inhibit TH reduction, the enzyme that catalyzes L-DOPA (L-dihydrophenylalanine) synthesis from tyrosine in the dopamine biosynthetic pathway [139]. In MPTP C57/BL mice, pretreatment of EGCG decreases α-synuclein expression and hinders neuronal death by increasing Bcl-2 and lowering Bax expression. It also provided neuroprotection by boosting striatal protein kinase C-α (PKC-α) [140]. In human NB SH-SY5Y cells, EGCG increases the expression of PKC, resulting in Bad degradation [141]. Activated PKC promotes neuronal survival by stimulating ERK and JNK [142]. EGCG also prevents neurodegeneration in CHO cells expressing dopamine transporters (DAT) by inhibiting MPP+ absorption and transfer to presynaptic dopaminergic neurons [143]. COMT inhibitors block conversion of L-DOPA to 3-O-methyl dopa and are used in junctions to treat PD. EGCG has been demonstrated to inhibit COMT, both in vivo and in vitro, preventing further methylation of L-DOPA, suggested to be given in combination with other medication to improve their availability and efficiency in the brain (Figure 5) [144]. In addition to upregulating dopamine conversion, EGCG inhibits MAO-B in aged rat brains, indicating its multi-potential function in the prevention of PD [145]. Iron accumulation in the substantia nigra is regarded to be a pathogenic feature of PD. When the BBB is disrupted and iron transport and storage mechanisms fail, the iron gathers in brain, which causes oxidative stress, aggregation of α-synuclein, neuro-inflammation, and ultimately cell death [146]. Iron chelation therapy is being studied as a therapeutic approach for treatment of PD. Green tea polyphenols’ potential as iron chelators, as well as other neuro-rescue characteristics, may aid to alleviate Parkinson’s disease (Figure 5).

#### 2.5.3. Huntington’s Disease (HD)

Huntington’s disease is initiated by repeated enlargement of unstable polyglutamine (poly Q) within the first exon of the IT-15 gene, producing Huntington (htt) protein [147]. The presence of htt fibril aggregates causes progressive damage to striatal and cortical neurons and also produces neuronal inclusions with aggregated htt protein [148]. Chorea, marked by fidgety movements, is a common symptom of HD. Tetrabenazine (TBZ) has been proven beneficial for chorea treatment as it reduces dopamine levels by suppressing the vesicular monoamine transporter 2 (VMAT-2) in the central nervous system. Few antipsychotic medications have also been used for such purposes in the past [149]. Choline esterase inhibitors (Rivastigmine) have been demonstrated to improve cognitive functions in progressive HD-related dementia patients; however, more placebo-controlled trials are required [150]. In the last few years, most research has focused on compounds preventing mutant htt aggregation. EGC and other green compounds are powerful inhibitors of htt1 aggregation. In vitro HD models were used to demonstrate that green tea polyphenols can regulate the early phase of polyQ growth, therefore impeding development of amyloid fibril processes. The potential benefits of EGCG and its derivatives against HD have been reported in many animal studies [151]. EGCG inhibits polyQ aggregation and protects neuronal cells expressing mutant htt protein [152]. Alterations in the lipid content of cellular and subcellular membranes also cause amyloid protein accumulation. It also had a better affinity for phospholipids than non-mutated HTT, causing changes in phospholipid double-layer stability. Beasley et al. [95] investigated the effect of lipid vesicles on EGCG or curcumin to modulate htt, lipid linkage, and aggregated htt. Because EGCG inhibits amyloid formation in the presence of lipid vesicles regardless of how it interacts with the membrane environment, it is suggested that EGCG could be used to treat HD and other disorders where protein aggregation is altered and coupled to amyloid deposition. A clinical trial examining the effect of the maximum routine dose of EGCG (1200 mg) on cognitive functioning of HD patients indicates intriguing preliminary findings; however, more human clinical trials are required to determine EGCG effects as a therapeutic candidate for HD treatment.

#### 2.5.4. Multiple Sclerosis (MS)

Multiple sclerosis is a chronic neurodegenerative auto-immune disease characterized by local lymphocytic infiltration, causing inflammation and demyelination of neurons as well as astroglial proliferation with neuronal injury [153]. It can produce a wide range of neurologic symptoms, including sensory impairments, movement issues, and impaired vision. The most prevalent clinical form of MS is RRMS (relapsing–remitting multiple sclerosis), distinguished by relapse with clinical manifestation along with partial or complete recovery [154]. Yet, there is no definite cure for MS. However, many disease-modifying treatments (DMT) for reducing relapses and disease progression have been reported [155]. Many studies have studied DMT and EGCG in the experimental autoimmune encephalomyelitis (EAE) model, specifically used for MS research. Inflammatory infiltrates and the onset of disease were significantly delayed [99]. Other research examined EGCG’s effects in cuprizone-induced MS animals. EGCG enhanced oligodendrocyte transcription factor 1 and remyelination-related proteolipid protein in the cerebral cortex [100,156]. In both animal models, EGCG therapy improved the mice’s clinical symptoms or molecular pathways. Few studies have investigated catechins’ influence on human populations, chiefly on RRMS. Bellman-Strobl et al. [157] conducted an analysis of EGCG + GA in 122 RRMS patients. When compared to the placebo, the 800 md/day oral EGCG + GA dose for an 18-month duration revealed no significant improved in MRI and clinical activities. A double-blind clinical experiment was conducted to investigate the antioxidant therapy mechanism in MS patients and showed that RRMS patients had lower NOX levels in CD11b+monocytes when given GA + 600 mg EGCG. The daily dose of 600 mg of EGCG in RRMS patients for 12 months has been reported to increase muscle metabolism during exercise [158]. Lovera et al. [103] conducted a study to assess the efficacy of polyphenon E (green tea extract containing 50% EGCG) in patients suffering from MS. They discovered that 800 mg EGCG enhances levels of N-acetyl aspartate in the brain, indicating a neuroprotective effect. However, due to abnormal liver function tests, the dose had to be stopped in five out of seven patients, concluding that polyphenon E may have an increased risk of hepatotoxicity. Other researchers assessed the EGCG treatment safety and determined that it was a safe drug, with comparable side effects in placebo as well as treatment groups [157,159]. In addition to EGCG, polyphenon E contains other polyphenols containing low quantities of caffeine that are metabolized in the liver. Overall, catechins’ effectivity in MS patients is still unclear.

#### 2.5.5. Amyotrophic Lateral Sclerosis and Frontotemporal Dementia

Amyotrophic lateral sclerosis (ALS) is a less common neurodegenerative disease, characterized by progressive loss of upper and lower motor neurons, which leads to weakness in limbs and swallowing and speech difficulties [160]. The progression of the disease ultimately results in paralysis and death from respiratory failure [161]. Frontotemporal dementia (FTD) is also associated with changes in the temporal and frontal lobes that manifest changes in personality, language, behavior, and motor skills [162,163]. EGCG has been reported to have a protective role in ALS in protecting motor neuron cells from mitochondrial damage and oxidative stress [164]. Oral EGCG supplements significantly slow the onset of symptoms, improve motor function, and expand lifespan [165,166]. EGCG upregulates the PI3K/Akt signaling pathway, which mediates GSK-3 activity, increasing neurofibrillary tangles and neuronal death [164]. An increase in PI3K/Akt and a reduction in death signals, including caspase-3, cleaved PARP, and cytosolic cytochrome c, are observed in ALS animals, which supports these findings [165]. On microglia and astrocytes, EGCG also possesses anti-inflammatory and anti-oxidant properties [166]. Furthermore, despite its presumed chelating properties, it has little effect on iron metabolism [167]. In FTD, inhibition of tau filament formation was observed for ECG but not for EC [168].

#### 2.5.6. Fetal Alcohol Spectrum Disorders (FASD)

Alcohol has many adverse effects on CNS, including neuro-immune dysregulation, neurotransmitter problems, epigenetic modification, as well as oxidative stress [169]. Chronic intake of alcohol leads to cognitive impairment, with loss of axons, atrophy of white matter, and brain cell demyelination [170,171]. The rat brain analysis revealed an association between alcohol consumed and degree of white matter atrophy [172]. FASD referred to the group of disabilities as the result of prenatal exposure to alcohol, marked by neurobehavioral dysfunction, dysmorphology of the face, and stunted growth [173,174]. To date, FASD and other alcohol-related changes have no treatment, and the traits remain throughout life; however, early intervention, including developmental therapy or behavioral interventions, may resist certain disorders’ and disabilities’ development and progression [175,176]. The potential of catechins to permeate various organs and their anti-oxidative effects bring them to the table for FASD treatment options [177]. In the murine model, 50 to 100 mg/kg EGCG treatment has been observed to increase glutathione and SOD levels, along with lipid peroxide and NO levels reduction in neonates [178]. Pregnant mice administered 400 mg/kg EGCG reported a decline in production of H_2_O_2_ and malondialdehyde (MDA) [179]. On the other hand, 30 mg/kg EGCG treatment for prenatal alcoholic exposure (PAE) resulted in N rf2 reduction [180]. As EGCG can cross the BBB, it has been shown that it can improve the brain development of a fetus impaired by ethanol [177]. In an in vitro rhombencephalic-neuron-cultured model from the rat fetus, EGCG prevents ethanol-induced neuronal death [181]. It also decreases the expression of NFB and caspase-3 in a rat model [178]. Microcephaly and PAE-related cognitive deficits may be prevented by reducing apoptosis. In addition to its antioxidant and anti-apoptotic properties, EGCG has been shown to reduce the ethanol-induced inflammatory response (decreased interleukin-1 and tumor necrosis factor-α levels) [148]. A study conducted on PAE mice showed that a daily 30 mg/kg EGCG dose increased expression of neuronal nuclei (NeuN) and doublecortin (DCX), which could help in preventing the loss of mature neurons and the delays in maturation due to ethanol. EGCG upregulates BDNF and glial fibrillary acidic protein (GFAP), thus compensating for the early astrocyte differentiation and neural plasticity abnormalities caused due to maternal alcohol consumption [180]. The tea catechins, including EGCG, EC, and catechins, have been observed to inhibit DNMT1-mediated DNA methylation in in vitro models [182]. EGCG effectivity against FASD DNA methylation was not found in the literature, so it would be informative to observe the effect of catechins on the DNA methylation pattern of fetal cells. Finally, the aforementioned disruptions in embryonic neurodevelopment result in behavioral and cognitive abnormalities, including impaired learning and memory having been observed to be ameliorated via EGCG treatment [104]. It is interesting to note that DS and FASD have many similar traits, including craniofacial dysmorphology, cognitive and behavioral disorders, and growth deficiencies. DYRK1A inhibition has been shown to aid neuronal plasticity in patients suffering from Down syndrome, suggesting that this molecular pathway should be investigated in FASD disease.

#### 2.5.7. Down Syndrome (DS)

Down syndrome is a genetic disease related to intellectual disabilities, caused by chromosomal 21 third copy, characterized by typical facial traits, including epicanthus, flattened face, up-slanted eyes, and hypotonia. The cognitive abnormalities in Down syndrome range in severity owing to the imbalance between huge synaptic suppression in the hippocampus and excessive cerebral cortex activation [183]. DS may cause a lack of flexibility in reaction to the environment, resulting in failure to adapt to a new situation. The major outcomes are changes in synaptic structure, which affects the information processing and storage capacity of brain networks. The neuropsychological traits include prominent hippocampal-dependent deficits impacting spatial memory, and reduced cognitive abilities [184]. EGCG, because of its pro-cognitive effects, has been recommended for Down syndrome [185]. EGCG also suppresses metalloproteinase 9 (MMP9), which is dysregulated in DS [186]. DYRK1A is over-expressed in Down syndrome and is considered to be the major source of cognitive dysfunction in DS patients. Researchers found that different doses of EGCG are associated with inhibition of DYRK1A in the brain. Thus, DS treatment can target the active DYRK1A concentration to improve behavioral issues via the GABAergic and glutamatergic pathways [187,188]. Environmentally enriched EGCG treatment in Ts65Dn mice has shown to improve memory and learning. A 42 mg/kg/day EGCG dose has also shown to retrieve deregulation of phosphoprotein in the hippocampus, reverse the kinome deregulation process, and restore the epigenetic profile. Because of all these potential pathways, green tea derivatives may enhance cognition [189,190,191,192]. EGCG therapy restores mitochondrial bioenergetics and biogenesis, which were significantly reduced in Down syndrome. It enhances proliferation of neuronal progenitor cells and neuronal plasticity and inhibits ROS production [193,194]. It is possible that neonatal EGCG therapy is more effective in reducing DS impairments. However, long-term EGCG effects on hippocampus physiology are still not proven [195].

## 3. Conclusions

To date, few therapeutic strategies for treating neurodegenerative diseases are available, so it is critical to discover novel preventive and therapeutic neuroprotective agents. Catechins appeared to have neuroprotective effects, as evident from both in vivo and in vitro studies against Alzheimer’s, Parkinson’s, multiple sclerosis, Huntington’s disease, and other neurodegenerative diseases. Catechins, especially EGCG, the most commonly used catechins, possess antioxidant, anti-inflammatory, anti-apoptotic and neuritogenic, and metal chelation characteristics, along with the ability to trigger diverse molecular mechanisms, such as lipid peroxidation inhibition, induce metal-chelating effects on amyloid-B, and α-synuclein fibrillation, inhibiting amyloid-β fibrillation. They produce anti-inflammatory effects by regulating the levels of inflammatory markers, including TNF-α, NF-kB, IL-6, IL-1 NFB, and NO, increasing antioxidants, such as GSH, SOD, and CAT, and decreasing lipid peroxidation by increasing Nrf2 protein expression. Moreover, catechins have the ability to cross the blood–brain barrier, which makes them feasible neuroprotective candidates against neurodegenerative diseases. However, more research, particularly clinical trials in humans, is required to understand catechins’ involvement in various biochemical pathways associated with neurological diseases. Future studies should examine regulation of neurological mechanisms and targeted brain regions where neuronal failure occurs, as well as bioavailability and optimal dosage for the best outcomes with no side effects and toxicity aspects regarding catechins consumption. Cognitive training along with pharmacological therapies would open up a world of possibilities for reversing the neuronal degeneration observed in such disorders.

## Figures and Tables

**Table 1:** Neuroprotective effect of catechins in various neurodegenerative diseases.

Objective	Experimental Model	Disease	Outcome	References
EGCG	In vitro	Alzheimer’s	Inhibition of Tau aggregation and oxidation	[94]
EGCG	In vitro	Huntington disease	Inhibitory effect on htt aggregation	[95]
CAT	Rat model	Parkinson disease	Improve rotational behavior, locomotion, and memory	[96,97]
EGCG	Rat model	Alzheimer’s	Decrease oxidative stress and improve cholinergic synaptic and mitochondrial functions	[98]
EGCG	Mice model	Multiple sclerosis	Decrease onset of disease and clinical severity. Reduce inflammatory infiltrates. Increase Olig 1 expression	[99,100]
Green tea	Drosophila model	Huntington disease	Green tea consumption may modulate symptoms	[101]
EGCG	Human studies	Multiple Sclerosis	Improve muscle metabolism, counteract NOX overactivation, decrease plasma NAA levels	[102,103]
EGCG	Human studies	Down syndrome	Improve visual recognition memory	[104]
EGCG	Human studies	Alzheimer’s	Low prevalence of cognitive impairment, decrease oxidative stress, and lipid peroxidation	[105,106]
